# The microglial immunoreceptor tyrosine‐based motif‐Syk signaling pathway is a promising target of immunotherapy for Alzheimer's disease

**DOI:** 10.1002/ctm2.1200

**Published:** 2023-02-11

**Authors:** Shoutang Wang, Marco Colonna

**Affiliations:** ^1^ Department of Pathology and Immunology Washington University School of Medicine St Louis Missouri USA

1

Alzheimer's disease (AD) is the most common form of dementia in the elderly.^1^ Age is the greatest risk factor for AD; thus, with the global increasing age of the population, the significance of this health problem will continue to escalate. The pathogenic mechanisms underlying AD are multifactorial and remain incompletely understood. AD can be classified into two subtypes: autosomal dominant AD (ADAD) and sporadic AD. ADAD represents less than 1% of all cases: it is caused by mutations in *Amyloid precursor protein* (*APP*), *Presenilin 1* (*PSEN1*), or *Presenilin 2* (*PSEN2*) genes, which control the proteolytic processing of the APP into the amyloid‐β (Aβ) peptides.^2^ Genome‐wide association studies have revealed that the genetic risk factors of sporadic AD are more diversified and include, among others, genes controlling microglial activation, such as immune receptors (*TREM2*, *MS4A4A*, and *CD33*), signaling intermediates (*PLCG2* and *INPP5D*) and growth factors (*IL34*).^3^ For instance, a partial loss‐of‐function TREM2 missense variant, *R47H*, significantly increases AD risk, whereas decreased AD risk is correlated with a gain‐of‐function *P522R* variant of PLCG2, a phospholipase Cγ family member that is a downstream signaling effector of TREM2. Therefore, a potential AD therapy based on microglia activation has raised broad attention.

Microglia, the brain resident macrophages, respond to Aβ deposition by forming a barrier that attenuates propagation and toxicity of Aβ in the early stage of AD. Transcriptional analysis has demonstrated that during this process microglia convert from homeostatic microglia to disease‐associated microglia (DAM) through a transitional state (TM). Moreover, microglia around plaques proliferate. Our previous results revealed that TREM2 deficiency in AD mouse models restricts the ability of microglia to surround Aβ plaques,^4^ proliferate,^5^ and convert to DAM.^6^ These defective microglia response leads to greater neuritic dystrophy adjacent to Aβ plaques. Based on this role in sustaining microglia response to Aβ, TREM2 is currently explored for antibody‐mediated immunotherapy.^7^, ^8^ However, TREM2 is just one of the receptors that activate microglia through the immunoreceptor tyrosine‐based motif (ITAM) signaling pathway, which is mediated by the protein tyrosine kinase SYK. This kinase phosphorylates and activates various downstream effector pathways, such as the PI3K‐AKT‐mTOR and the PLCγ2‐Ca^2+^ pathways. Conversely, SYK‐mediated phosphorylation of GSK3β inactivates GSK3β, releasing β‐catenin from inhibition thereby inducing proliferation. In our recent publication in *Cel* *l*,^9^ we examined the impact of the SYK pathway on microglia by generating a *Syk* conditional knockout mouse that selectively lacks SYK in microglia and crossing this mouse with the *5xFAD* mouse model of Aβ pathology. We found that constitutive SYK deficiency impaired microglia's ability to respond to Aβ plaques, resulting in increased Aβ accumulation, neurite dystrophy, and memory deficits. Moreover, induction of SYK deletion at a late stage of the disease reversed microglial clustering around Aβ plaques, suggesting that SYK is required not only for a generation but also for the maintenance of microglia responses to Aβ.

Microglia express a large network of germline‐encoded activating receptors that transmit signals through SYK. Integrins that control adhesion and migration also signal through SYK. Thus, we expected that SYK deficiency would also affect microglial development or homeostasis. However, we observed no obvious defects in microglial numbers, morphology, and phenotype in the absence of Aβ, suggesting that SYK signaling is redundant for microglia development and, self‐renewal. Another unexpected observation of our study is that although many ITAM‐signaling receptors transmit signals through SYK, the amyloid pathology observed in SYK‐deficient mice was not more severe than that observed in TREM2‐deficient mice, suggesting that TREM2 is the predominant microglial receptor signaling through SYK, whereas other receptors may be expressed in low amounts or may not be engaged by endogenous brain ligands. This may be the case of the C‐type lectin domain containing receptor CLEC7A, which was originally shown to enable macrophage recognition of the fungal molecule β‐glucan but is also expressed in microglia around amyloid plaques, even though it may not be engaged by Aβ.

Although TREM2 and SYK deficiencies seemed to have a similar impact on microglial responses to Aβ, single‐cell RNA‐seq of microglia performed in our study did reveal an important difference. Lineage trajectory analysis of single‐cell RNA‐seq data showed that both TREM2 and SYK deficiencies impaired the final differentiation of DAM; however, the TM population prodromal to DAM was mainly affected by TREM2 deficiency, whereas SYK deficiency has minimal impact. Thus, this TM of microglia is TREM2‐dependent but largely SYK‐independent. Moreover, SYK deficiency, in contrast to TREM2 deficiency, left microglia proliferation unaffected. These data suggested that TREM2 activates microglia not only through an SYK‐dependent but also an SYK‐independent pathway (Figure 1).

**FIGURE 1:**
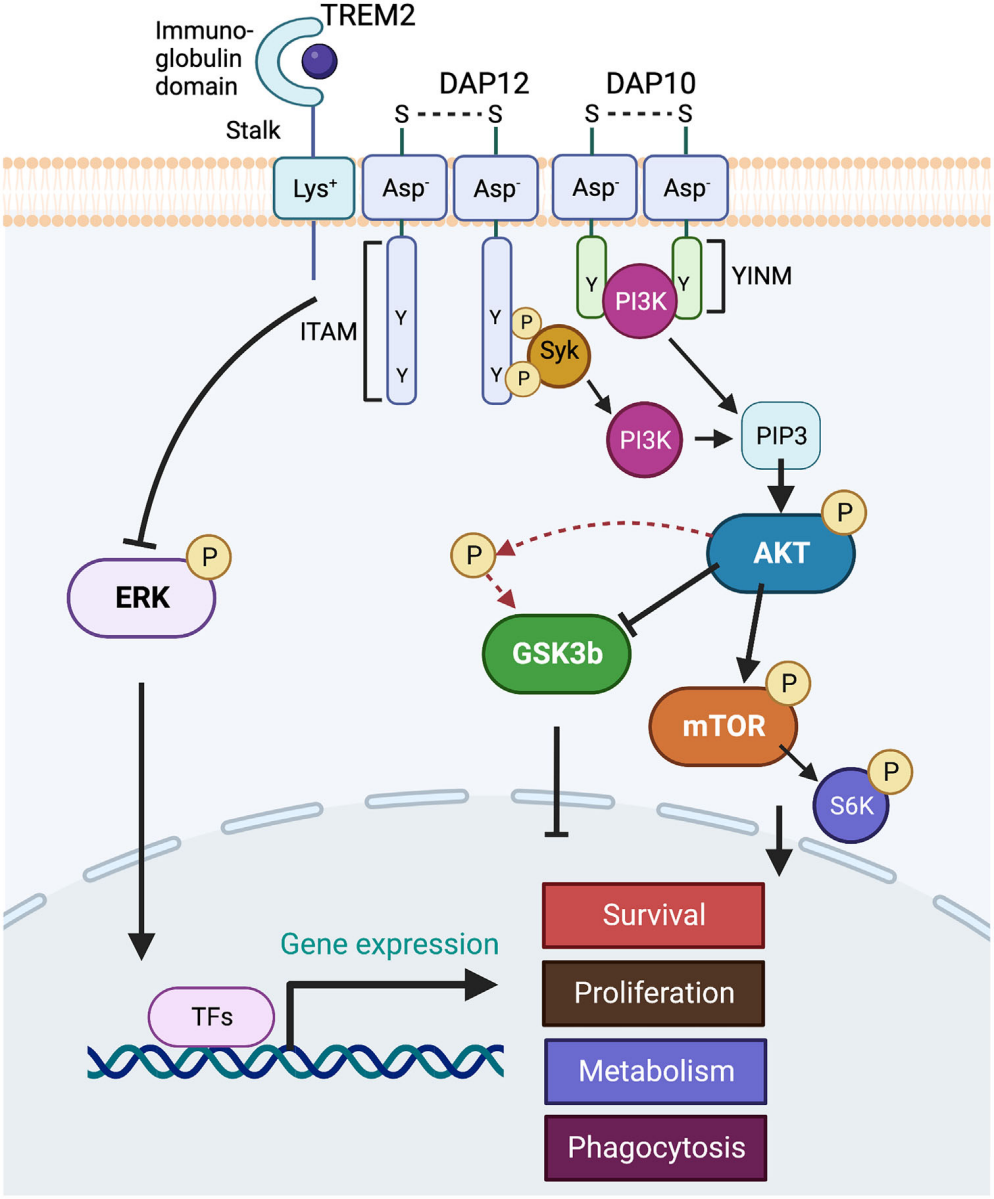
TREM2 transmits intracellular signals through the DAP12‐Syk and DAP10‐PI3K pathways. TREM2 associates with the transmembrane adaptors DAP12 and DAP10 to transmit intracellular signals. DAP12 contains cytoplasmic immunoreceptor tyrosine‐based activation motifs (ITAM), which recruit the non‐receptor spleen tyrosine kinase (SYK) that promotes tyrosine phosphorylation and activation of multiple downstream mediators. TREM2‐DAP12 can also inhibit the ERK signaling pathway that does not require SYK; DAP10 contains a cytoplasmic YxNM motif (YINM) that directly recruits PI3K. Both Syk and DAP10 signaling pathways activate mTOR while phosphorylate and inactivate GSK3β via AKT, which modulates effectors required for survival, cell cycle progression, metabolic activation, and phagocytosis.

Indeed, TREM2 is associated with two distinct signaling adapters, DAP12 and DAP10. DAP12 contains a cytoplasmic ITAM that recruits SYK. In contrast, DAP10 contains a cytoplasmic YXNM motif that directly recruits the PI3K and the adapter GRB2 that initiates MAPK activation. Comparison of single‐cell RNAseq data of DAP12‐ and SYK‐deficient microglia showed similar preservation of TM and proliferating microglia, which conversely wane in TREM2‐deficient microglia, corroborating that TREM2 partially signals through a DAP12‐SYK independent pathway that may be DAP10‐mediated. Consistently, DAP10 deficiency in microglia reduced AKT and GSK3β phosphorylation, microglia proliferation, and phagocytosis. These defects were exacerbated by an SYK pharmacological inhibitor, suggesting complementarity of the DAP10 and SYK pathways. Both DAP10 and SYK defects were rescued by a GSK3β inhibitor, highlighting the importance of GSK3β inactivation downstream of DAP10 and SYK signaling in promoting microglia activation and further supporting the beneficial effects of GSK3β inhibitors that have been reported in neurodegeneration.

Can we harness these observations for AD therapy? Since CLEC7A can activate SYK but is most likely not engaged in the brain during AD, we hypothesized that we could use a CLEC7A antibody as a surrogate ligand to induce SYK activation in an AD model with defective microglia. Indeed, engagement of CLEC7A by systemic injection of an anti‐CLEC7A antibody was able to offset the defect of microglia activation in vivo in mice carrying the *R47H* hypofunctional variant of TREM2. It is conceivable that many innate immune receptors other than CLEC7A that activate SYK can similarly sustain the magnitude and duration of microglia responses to protein aggregates, dead neurons, and myelin debris associated with AD. Therefore, antibodies and small molecules activating the broad range of microglial receptors that trigger ITAM‐SYK signaling are potential candidates for AD treatment.^9^, ^10^

## CONFLICT OF INTEREST STATEMENT

Marco Colonna has patents pending on TREM2 and CLEC7a and is a member of the advisory board for Vigil Neuro.
